# Free to choose: Mutualist motives for partner choice, proportional division, punishment, and help

**DOI:** 10.1371/journal.pone.0266735

**Published:** 2022-05-05

**Authors:** Chien-An Lin, Timothy C. Bates

**Affiliations:** Department Psychology, The University of Edinburgh, Edinburgh, United Kingdom; Academia Sinica, TAIWAN

## Abstract

Mutualism–the disposition to cooperate in ways that benefit both actor and recipient–has been proposed as a key construct in the evolution of cooperation, with distinct adaptations for 1) partner choice, 2) division, 3) punishment, and 4) helping. However, no psychological validation of this 4-fold psychological structure exists, and no measure of the trait is available. To fill this need, in two pre-registered studies (total N = 902), we: (A) Develop and administer items assessing each of the four mutualist adaptations; (B) Show good fit to the predicted four factor model; (C) Demonstrate reliability and stability across time; (D) Evidence discriminant validity from existing constructs, including compassion and utilitarianism; (E) Establish external validity by predicting proportional choices in catch division, opposition to partner coercion, and reduced support for redistribution; and (F) Replicate each of these findings. Jointly, these results support the validity of mutualism, including a motive to maintain the freedom to choose, and provide reliable scales for use in integrating, further developing, and applying mutualism.

## Introduction

Partner choice mutualism has been proposed as a key construct in the evolution of cooperation, specifying motives for proportional division, proportional punishment, and helping behavior [1]. This theory suggests that under a partner choice regime, counter-party failure to share the benefits of cooperation proportional to contribution leads actors to switch partners, selecting against partners who fail to respond mutualistically. Under free partner choice, selection favours adaptations promoting i) proportional sharing of benefits, ii) proportional punishment of crimes, and iii) helping when benefits to others are disproportionately greater than costs to the actor [1]. Despite the obvious potential importance of this suite of adaptations for understanding moral behavior, no psychological test of the 4-fold structure has been made, and no psychological measures have been developed to assess it. Here in two studies (total n = 902), we develop a questionnaire measure, validate the predicted 4-factor psychometric structure, test its reliability across time, test discriminant validity from existing constructs, and test its external validity. Finally, we replicate each of these findings in independent data. Before presenting these studies, we background the theory and four psychological mechanisms underlying mutualism.

### The mutualism model

Within Hamilton’s framework of benefits and costs between an actor and recipient [2], mutualism falls into the quadrant in which both actor and recipient benefit. Early work in this area focussed on partner control mutualism, exemplified in the iterated Prisoner’s Dilemma and strategies such as Tit-for-Tat designed to maximize benefits by controlling partner behavior [3]. Recently, however, research has highlighted the distinctive adaptive benefits of “partner choice” forms of mutualism, which offer the option to respond to non-proportional sharing of benefits by switching partners rather than seeking to control a non-mutualist partner [4]. Thus, while both partner control and partner choice involve a motivation to assure outcomes proportional to some factor, under partner choice regimes actors have an “outside option”– to choose a new partner.

Baumard, André, and Sperber [1] proposed that this freedom to choose enabled key achievements in human morality, detailing a suite of three specific adaptations involving sharing benefits, punishment, and helping others, enabled by partner choice. Actors are selected to reject exploitative partners whilst maximizing their own benefit by attracting more generous partners. This creates a selective pressure against both emitting and accepting exploitative behavior, instead favouring proportional intuitions. Individuals faced with a cheating or otherwise non-mutualist partner are incentivised to begin a new cooperative relationship with a different, potentially more mutualistic partner. Switching to a new partner creates social selection, with mutualist actors leaving greedy partners, and fostering an increase in pairings between those willing to cooperate mutualistically. This in turn creates selective pressure against exploitative partner behaviors [1]. Competition for partners can, thus, select for dispositions one might expect if a mutually agreeable contract was in force between free agents. This aspect of “fair” behaviors being of a kind which would be mutually agreeable should they have been passed as a contract between cooperators has been noted in comparative research [5] as well as in philosophy [6]. A key question, then, is what form, precisely, should these fair disposition(s) take? This is discussed next.

### Mutualist motives: Specifying the phenotype

As noted above, Baumard, André, and Sperber [1] specify a suite of three adaptations which they predict will be selected under a partner-choice regime: i) A motive to share the benefits of cooperation in proportion to the contribution made, termed “Proportional Sharing”; ii) A motive to punish wrong doers in proportion to their crimes; and iii) A motive to provide help when the benefits to the receiver are disproportionately greatly than the costs to the giver. In addition, we expect that mutualists have a motivation to use, maintain and protect their option for partner choice, and we hypothesise that all four motives will covary with each other, reflecting their joint dependency.

Partner choice creates a selective pressure to condition rights to the fruits of joint labour on contribution–“Proportional Sharing”–as follows. In populations of individuals differing in effort and talent, freedom to choose partners creates a distribution of interaction benefits for individuals. Outcomes are necessarily maximized for individuals if they enter the interaction providing the greatest available benefit. If contributing more than the average partner, the mutualist will gain by choosing partners motivated to credit this excess contribution to them, increasing their yield. Even when contributing less to a given interaction, the mutualist’s willingness to accept proportionally less allows them to attract and retain high-quality partners, allowing them to share (albeit proportionally) in more successful outcomes and increasing their pool of willing cooperators essential when partnership is voluntary.

Regarding punishment, mutualism predicts this proportional punishment motive will emerge under partner choice in order to deter cheating by assuring that this will not pay in their relationships. The purpose of the motive, then, is not to punish the wrongdoer at a cost to the punisher, but rather to minimise future partnering with wrongdoers. Calibration to the costs of the wrongdoing minimises punishment costs of inflicting the punishment [1]. At the low end, punishment consists in simply ceasing interaction. Not interacting with a wrongdoer has benefits for the mutualist, who avoids costs incurred with non-mutualist partners who fail to share proportionately or to provide beneficial help, replacing these costs with benefits from their interactions with others not observed to do wrong, and therefore more likely to offer mutualist benefits. Such low-cost ostracism is observed widely in foragers [7, 8]. For more severe wrongdoing, the mutualist’s infliction of proportional punishment is selectively advantaged by deterring the wrongdoer from future aggression [9]. Punishment aimed at protecting the victim herself by reducing future aggression [10] also benefits the mutualist. By contrast, escalated retaliation aimed not at protecting the victim from this and other wrongdoers but rather at equating outcomes for wrongdoer and victim (eye for an eye) creates potential for interactions which can yield additional, disproportionate costs [11]. The mutualist’s proportional punishment, then, disincentivises wrongdoing and minimising interactions with wrongdoers, while avoiding the high costs of retribution. This provides benefits under which the genes for this motive can be selected for over genes leading to either retaliation or capitulation.

Evidence for proportional punishment has been found in anthropological observations of displays of displeasure in cases where compensation either greatly exceeds or falls beneath the level of harm experienced by a victim [12]. Mutualists are also expected to punish more potently when either they are themselves the victim, costs of punishment can be mutualised (shared, e.g. via group enforcement) or when costs are minimal allowing low-cost restoration of the victim. In this case, mutualists are reinforced for calibrating their punishment at levels which are restorative, but do not go so far as to commit an injustice to the wrongdoer. By causing wrongdoers to compensate victims the unfair benefit they sought is removed by the costs of restoration, multiplied by any chance of going un-detected, thus removing the systemic incentive for wrongdoing without triggering a warranted sense of injustice to the wrongdoer(s).

The third dimension of mutualism, perhaps surprisingly, predicts that partner choice can select for a helping adaptation, such that help will be provided where this has benefits to others which greatly exceed the costs to the helper (i.e., which is low-cost relative to the sum of its benefits to recipients) and discourages help which is of little benefit but of high cost. This help ranges from turn-taking in bearing severe risks, to niceties such as holding opening doors for those following close behind, to high value information shared at low cost–for instance a mentor offering a recipient invaluable advice which costs them only a brief communication–to name but a few. By definition, this aid is a powerful net benefit to members of any community in which mutualist helping occurs. Because helping (and failure to help) is highly visible, cheaply stored as personal tokens in the minds of other group members, and with tokens cheaply communicated throughout the social network, a moral helping intuition can be selected for by mutualists choosing the community with which they interact and shunning actors and communities of people who are observed to not provide these helping behaviors, thus depriving non-helpers of the (by definition) much larger, benefits of helping from other mutualists [1]. Examples would include teams, neighbourhoods, cliques, etc. within which people motivated to offer high value help are in a position to receive high value help from similarly motivated neighbours.

All the gains above are conditional on access to and use of outside opportunities (i.e. the freedom to choose partners). For this reason, and as a fourth and final adaptation, we predict that mutualists will not only show the three predicted motives for proportional sharing, punishment, and helping, but also will also actively use and defend their ability to freely choose partners, responding by moving away, investing work to defend this freedom of association and in this way avoid interactions in which the marginal benefit of their investment is lower than the average benefit they could gain elsewhere. Support for the value of this hypothesised preference for freedom to choose partners comes from, for instance, economic game studies [13–15], showing that switching to a new partner (“Walk Away” strategy) not only minimises interactions with cheaters but also maximises benefits of interacting by increasing the chances of meeting optimal co-operators [16]. Support for a preference among people to choose partners is also well supported. In anthropological studies, for instance, food exchanges among hunter-gatherers provide additional support for partner choice: hunter-gatherers avoid dealing with untrustworthy partners or cheaters, switching to other partners who are willing to share [17, 18].

In summary, while mutualism is an influential theory, with a suite of clearly defined motives, and concrete predicted external markers of discriminant validity in the domains of sharing, punishing, helping, and maintenance and exercise of freedom to choose who one cooperates with and who one excludes, the psychological structure has not been tested and no psychological measures have been developed to assess mutualism. Therefore, in study one, we developed candidate items based on the concepts introduced above, collected data, tested the fit of the proposed factor structure to these items, and tested the reliability, external and discriminant validity of the resulting scales.

## Study 1

In study 1, we set out to develop a measure of mutualism, to test the fit of the proposed four factor structure, and to test the reliability, and discriminant and external validity of the scales. A pool of items was generated based on Baumard, André, and Sperber [1]. Example items were sourced from the examples and language of this work, as well as from our own translation of the evolutionary background and behavioural expectations for partner switching, division, punishment, and helping based on the theory. Thus, to develop a scale to assess individual variation in the motive to switch partners, and to contrast this with the non-switching motive to control partners [e.g. 3] items such as “*If someone tries to free-ride on my efforts*, *I exclude them*”, “*If someone cheats me or treats me unfairly*, *I find someone else to interact with in future*” and “*I choose who I invest time resources and energy with rather than try and change them*” were generated. For proportional sharing of benefits candidate items such as “*Those who have contributed more should receive more*”, “*People should receive rewards in exact proportion to their contribution*” and “*Rights should be in proportion to duties*” were generated. For proportionality of punishment, items varied from the straightforward “*Punishment should be proportional to the crime*”, “*A week in jail for stealing an apple would be too long*” to “*Wrong doers should compensate their victims by an amount proportionate to the harm they have inflicted*”. Finally, for proportional helping, example items again varied from “*I help others whenever it is easy for me to do so*”, “*I expect others to help me when this is low cost to them*” to “*I help when it helps others much more than it costs me*, *like holding a door open*”.

This process led to a candidate pool of 41 items which were administered to a large sample along with validation measures described below. Based on mutualism theory, we predicted that four correlated factors would be identified, corresponding to the architecture of mutualism. To test this, a preliminary factor analysis extracting four factors was used to identify well-performing items (if any emerged) for each of the four prospective mutualism components. Guided by this, a structural model consisting of four factors under a hierarchical general mutualism factor was constructed. Internal reliability was tested using Cronbach’s alpha and test-re-test stability of the scales was assessed across a 5-month period.

## Validation measures

In addition to developing and testing the structure and reliability of potential mutualism scales, we wished to test the discriminant, external and incremental validity of the new scale. Demonstrating discriminant validity requires demonstrating independence of the trait of interest from competing measures which could plausibly play the role assigned to the trait of interest, but which are theoretically distinct. As mutualism is predicted to be an evolved fairness intuition assuring impartiality, defined as behavior which is mutually advantageous [1], we assembled competing measures of fairness, and motives proposed to account for cooperative behavior in other theories and from which mutualism must show discriminant validity if it is to be supported as a distinct account of motivations for proportional division.

Multiple accounts have emerged in social science to account for division and fairness [19, 20]. To test discriminant validity, we compared the mutualism scale to scales designed to explain division and distribution in the three-player two situation model [21] which assesses compassion [21], self-interest [21] and envy [22]. The second major measure chosen to evaluate discriminant validity was the Oxford Utilitarianism Scale (OUS), designed to account for intuitions regarding fair division under a utilitarian ethic of fairness or equality. The OUS consists of independent dimensions of Impartial Beneficence and Instrumental Harm [23]. Recently, research on consequentialist reasoning [23] has articulated two well-validated scales assessing a specific notions of fairness-as-impartiality in terms of two independent motives underlying utilitarian consequentialist decision making: impartial beneficence and instrumental harm [23]. Given the availability of these measures, we wished, therefore, to test association of mutualism to impartial beneficence. Baumard, André, and Sperber [1] predict that mutualism should be unrelated to utilitarian motives, describing these as “*not the kind of disposition one looks for in potential partners*” [1] and as orthogonal to mutualist notions of fairness. Note: At the time we pre-registered our expectations we construed the mutualist motives of sharing proportional to contribution rather than need and voluntarism rather than coercion in relations as possibly predicting not just independence from utilitarian motives, but an inverse relationship. This negative relationship expectation, however, does not follow from mutualism theory (and was not supported).

This brings us to the choice of external validation. To test this, in the first instance, we tested if mutualism predicted attitudes to sharing using vignettes in which subjects chose preferred sharing of food in imagined hunting situations. This scenario was chosen as being able to be used in other populations and because food sharing and distribution of large resource packages has been common since hunter-gatherer groups where such distributions can be so extensive that successful hunters and their families may make no extra dietary gain from their hunting ability [24]. Based on mutualist motives to keep rewards proportional to contribution, we predicted that mutualism scores would be negatively related to sharing with division into equal shares regardless of contribution.

As a second external validator of mutualism, we turned to what is perhaps the central contemporary socio-political divide–support for redistribution. This is a widely studied and robust outcome variable with important social impacts [21]. Support for redistribution via tax and welfare policy shows substantial variation [25] and is intimately connected to the allocation of goods in response need rather than contribution. As mutualism predicts that cooperation should be voluntary and rewards kept proportional to contribution, we predicted that mutualism should predict reduced support for redistribution.

In summary, we predicted that (1) The mutualism items would show a four factor structure; 2) That well performing scales in a well-fitting structural equation model could be extracted from the item pool; 3) These scales would be correlate under a general mutualism factor; 4) Mutualism would be reliable, and show temporal stability; 5) Mutualism would show discriminate validity from compassion, envy, self-interest, as well as impartial beneficence, and instrumental harm (testing also for a negatively association with the latter scales); 6) Mutualism would predict preferences for proportional sharing in a series of scenarios; 7) Support for external validity would be shown in a significant negative relationship to support for redistribution. Predictions and sample size were pre-registered on AsPredicted.org.

## Method

### Participants

A total of 400 participants were recruited using Prolific Academic (252 females, mean age 40 years (SD = 12.54)). This was influenced by minimum guidelines regarding subject to construct/item ratios of from 5–10 subjects per construct [26, 27], but greatly exceeded these. We pre-registered a criterion that subjects who completed the questionnaire less than 30 seconds would be excluded and no subjects met this criterion. Participants identified as White (n = 366; 91.5%), Asian (n = 18; 4.5%), Mixed (n = 10; 2.5%), Black (n = 4; 1.0%) and other (n = 1; 0.3%). Study procedures were approved by the Psychology Research Ethics Committee at the School of Philosophy, Psychology & Language Sciences in the University of Edinburgh (Ref No. 417-1718/6). All participants gave informed consent.

### Measures and procedure

Mutualism was measured with 41 candidate items targeting Partner Choice, Proportional Sharing, Proportional Punishment and Proportional Helping. All items used a Likert response scale coded from 1 (strongly disagree) to 5 (strongly agree). Items are listed in supplemental material. Internal and external, and incremental predictive validity analyses used additional measures which are detailed in the appropriate sections below.

Procedurally, the mutualism scale was administered after the other items, all items and scales were given in a fixed order. Participants were shown an Information Sheet about how we would keep their data saft and how to use them, and all participants were informed that they could choose to leave the study or delete their response at any time. Then participants read the information sheet and then were offered a consent form asking them if they consented to join this study voluntarily or not, before proceeding to the scales themselves. After participants completed the study, a debriefing sheet was shown explaining the details of this study and they were returned to prolific academic to verify their participation.

## Results

To test the four-factor hierarchical measurement model following the pre-registered hypothesis, and to select items for potential scales from the items pool, we first conducted a 4-factor analysis on all 41 items as a guide to item selection for the model building stage. We retained the first four items with the highest standardized item loadings in each factor, with the proviso that a minimum loading of .4 was required for the item to be retained. Based on these criteria 15 items were retained. For the “Proportional Punishment”, only three items were retained this being all the items of the pool which met the standardized item loading higher than 0.4 criterion.

**Fig 1:**
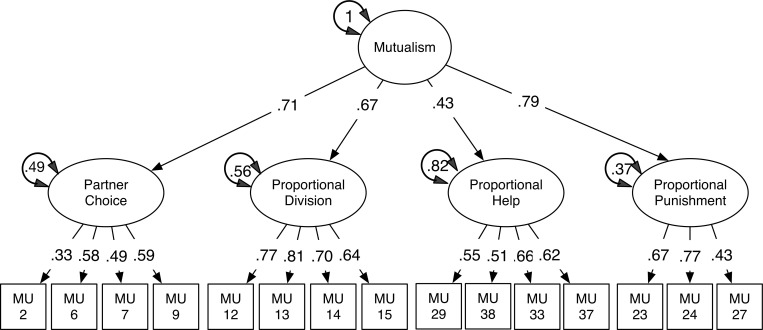
Well-fitting measurement model of mutualism, study 1.

We next fitted a four-factor structural equation model using the R package *umx* [28], constructed with each of the 15 retained items loading on one of four specific factors and with a hierarchical general mutualism factor above the four facets of mutualism. Maximum Likelihood (ML) estimation was used to estimate the model. A hierarchical four-factor model fit well, as predicted, the theoretical model and standardized parameter estimates are presented in Fig 1

This structural model of mutualism had good fit (χ^2^(86) = 180.26, *p* < .001), RMSEA = .052, CFI = .929. Almost all fit statistics of the Mutualism scale met recommended cut-off criteria [27, 29]. The questionnaire and its four scales as modelled, then, show good fit to observed data, and acceptable construct validity. This 15-item scale was used as our measure of Mutualism in subsequent tests of predictions. All items are shown in Table 1.

**Table 1:** Items of the final 15-item mutualism scale.

Original item	**Partner Choice**
MU2	If someone cheats me, I find a new, hopefully more cooperative, partner.
MU6	It is crucial that people can freely choose who they work and interact with—we shouldn’t be forced to associate with anyone or any group.
MU7	I choose who I invest time resources and energy with rather than try and change them.
MU9	It’s important that we can choose who we live near or trade with.
	**Proportional sharing**
MU12	Those who have contributed more should receive more.
MU13	People should receive rewards in exact proportion to their contribution.
MU14	People with the ability to produce more should receive more than others they are working with.
MU15	People who create more due to their greater talent or work should benefit exactly in proportion.
	**Proportional punishment**
MU23	Punishment should be proportional to the crime.
MU24	Wrong doers should compensate their victims by an amount proportionate to the harm they have inflicted.
MU27	Ten years in jail is not enough for murder.
	**Proportional helping**
MU29	I hold the door open for others.
MU33	I help when it helps others much more than it costs me, like holding a door open.
MU37	For things that I think of as almost costless, like holding a door or costs that don’t matter to me I do them.
MU38	I don’t like when people don’t do the little things to help others at almost no cost, like holding a door, or keeping their feet off other people’s furniture.

### Internal and test-retest reliability

To test the reliability of the new scale, we examined both the internal consistency of the overall scale, and conducted a 5-month test-retest reliability assessment. The Cronbach Alpha of the 15 item general scale was 0.73: an acceptable value [30]. To examine test-retest reliability, 5 months after subjects answered the initial mutualism candidate items, all participants were recontacted and invited to take part in a second study. The acceptance rate was 75.75%, with a total of 303 participants completing the re-test online (197 females, mean age 40 years, SD = 12.48). Examination of the internal consistency of the total scale again revealed good retest reliability (Cronbach Alpha = 0.78). Strongly supporting the stability of the mutualism scale (and trait), scores across this 5-month interval correlated at the limit of the reliability of the scale, suggesting scores are stable for retest (*r* = 0.72 (CI95% [0.66, 0.77], *t(301)* = 17.80, *p* < .001). Thus, the Mutualism scale comported itself well in terms of both consistency and test–retest reliability, with test-retest correlations comparable to the internal consistency of the scales.

### Criterion validity: Predicting social group attitudes

To examine criterion validity, we created scenarios involving sharing behaviour in a small group situation, to test if preferences expressed in these sharing scenarios were significantly predicted by mutualism scores. The scenarios created for this study assessed food-sharing behaviour in imagined small groups across 8 situational questions. In each of these, participants were asked to answer their reaction toward each situation as if they were living in a small tribe in primitive circumstances. Example items included “*Thinking about hunting with someone much better than you at hunting and who feels it is fair that they take proportionally more of the food most days*. *How much (if at all) would you try to get them to share more evenly with you*?” and “*How much do you agree with this statement*: *Letting others take an even share when this is more than they contributed is good for the group*” (full items in online matter).

**Fig 2:**
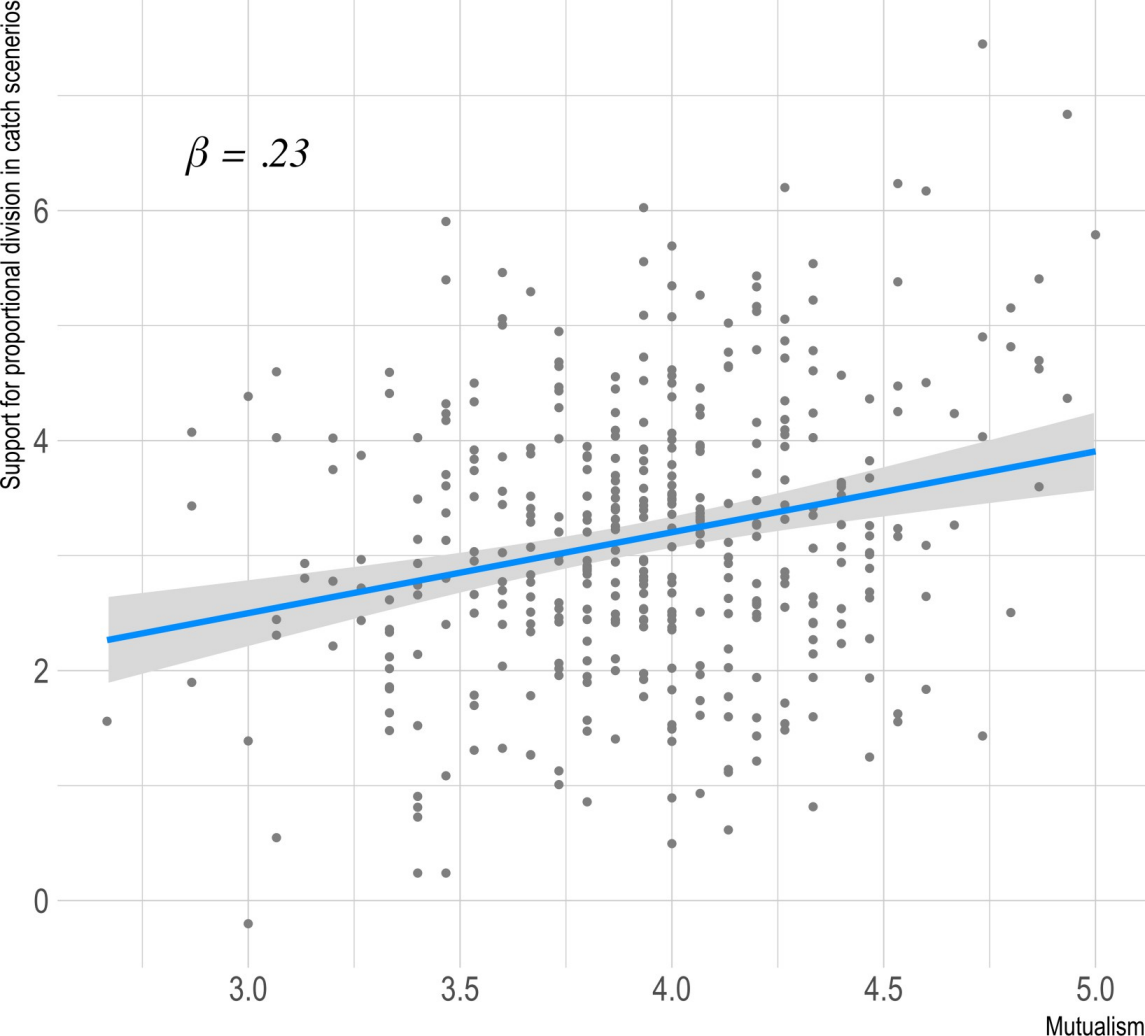
Association of mutualism with the sum of three catch-sharing scenarios (Study 1).

The hypotheses that mutualism would be associated with support for sharing proportional to contributions, and that mutualists would be averse to attempts at equal sharing when contributions differed were supported by our data. Support for equal division was significantly negatively correlated with the Mutualism scale (*r* = -0.13, *t(398)* = -2.60, *p* = .009). Likewise, believing equal catch division is preferable (*r* = -0.26, *t(398)* = -5.37, *p* < .001) and coercing others to share equally (*r* = -0.16, *t(398)* = -3.32 *p* < .001) related significantly negatively to mutualism. The association between Mutualism scale and the sum of three catch-sharing scenarios is shown in Fig 2. These results support the criterion validity of the Mutualism scale, with mutualists not only tending not to choose to share what they got equally, but also having aversion to equal catch division.

### External validity

Having shown that mutualism scores significantly predicted scenario-based preferences, we next sought to bolster this work by testing association of mutualism with actual support for proportional sharing, as expressed by the subjects with reference to their own society. To test this, we chose support for redistribution as a target variable. Support for redistribution is linked to behaviors with real-world validity, for instance in political voting patterns, and is well measured by reliable scales [31]. Attitudes toward redistribution were measured with the 11-item support for economic redistribution scale [21]. Example items include: “*Inequality in the distribution of wealth is unjust*” and “*Wealthy people should not be taxed more heavily than others*” (reversed). Each item used a Likert response scale from 1 (strongly disagree) to 5 (strongly agree). The Cronbach Alpha of economic redistribution in our sample was 0.89.

**Fig 3:**
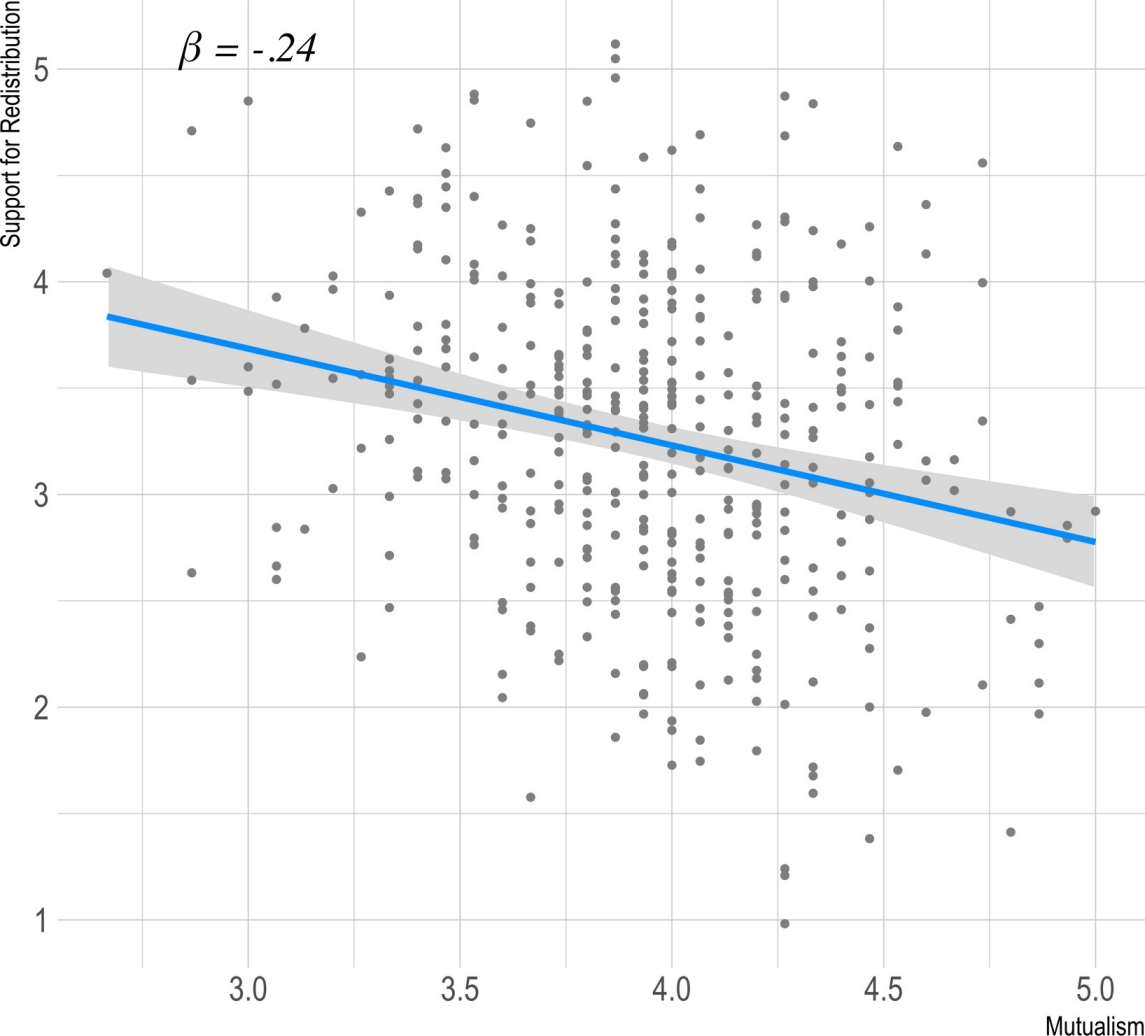
Graph showing effect of mutualism on support for redistribution in study 1.

We tested the prediction that mutualism would be negatively related to support for redistribution in a linear regression. Fig 3 shows the simple association of mutualism with support for redistribution, controlling for age and sex (*β* = -0.24 [-0.33, -0.14], *t(396* = -4.89, p < 0.001).

### Incremental validity

To test for incremental validity of mutualism in predicting support for redistribution, we included three known and replicated predictors of this outcome– compassion, envy, and self-interest [21, 32], to test that mutualism would remain significant, accounting for incremental variance in support for redistribution over and above these variables. Dispositional compassion was measured with the 10-item dispositional compassion scale. Items in this scale were selected from Goldberg [33] by Sznycer, Seal [21]. Examples include “*I suffer from others’ sorrows*” and “*I can’t stand weak people*” (reversed). Each item used a Likert response scale from 1 (very inaccurate) to 5 (very accurate). The Cronbach Alpha of this scale was 0.81 in our sample. Envy was measured with the 5-item Malicious Envy Scale from Benign and Malicious Envy Scale [22]. Items were scored from 1 (strongly disagree) to 6 (strongly agree), no items are reversed. An example item is: “*If other people have something that I want for myself*, *I wish to take it away from them*”. The Cronbach Alpha of Malicious Envy Scale in our sample was 0.83. Self-interest was measured with one item in this study: “*Imagine that a policy of higher taxes on the wealthy is implemented*. *What overall impact do you think the higher taxes on the wealthy would have on you*?”, with responses on a 1 to 5 scale: My own economic situation would 1: significantly worsen; slightly worsen; stay the same; slightly improve; 5 significantly improve.

Table 2 shows the results of a model predicting support for redistribution by mutualism, controlling for self-interest, compassion, and malicious envy. Mutualism remained highly significant (*t(393)* = -3.59, *p* < .001) and in the predicted direction with mutualism scores associated with reduced support for redistribution (beta = -0.14 (CI95% [-0.22, -0.07]). The prediction that mutualism would be negatively related to support for redistribution when compassion, envy and self-interest are controlled was tested using linear regression with these additional variables as covariates. Mutualism remained significant (*t(393)* = -3.59, *p* < .001), with an effect in the predicted direction (reduced support for redistribution of beta = -0.14 (CI95% [-0.22, -0.07]). As shown in Table 2, effects of compassion, malicious envy, and self-interest also replicated: (*t(393)* = 12.09, *p* < .001; *t(393)* = 6.74, *p* < .001), and *t(393)* = 6.39, *p* < .001, respectively).

**Table 2:** Regression models predicting support for redistribution.

Variables	Model 1	Model 2
Age	-.15 [-.24 .05] **	.00 [-.08 .08]
Sex	-.03 [-.13 .06]	-.07 [-.15 .00]
Mutualism	-.24 [-.33 -.14] ***	-.14 [-.22 -.07] ***
Compassion		.51 [.42 .59] ***
Envy		.28 [.20 .36] ***
Self-interest		.25 [.17 .33] ***
R^2^	.070	.403

*Note*. Effects are standardized regression coefficients [followed by 95% CI].

*** = *p* < .001

** = < .01

* = < .05.

### Discriminant validity

We examined discriminant validity among the three motives measured above via a correlation table. As seen in Table 3, the measures were largely independent, supporting their targeting of distinct motives. We also tested discriminant validity from utilitarian measures– a hard test as these also aim to measure fairness, but from distinct theoretical bases. We used the Oxford Utilitarianism Scale [23]. This 9-item measure consists of two subscales: Impartial Beneficence (OUS-IB) and Instrumental Harm (OUS-IH). All items are positively scored with responses coded from 1 (strongly disagree) to 7 (strongly agree). Example items include: “*It is just as wrong to fail to help someone as it is to actively harm them yourself*” (OUS-IB) and “*It is morally right to harm an innocent person if harming them is a necessary means to helping several other innocent people*” (OUS-IH). Cronbach Alpha of each subscale was 0.68 (OUS-IB) and 0.68 (OUS-IH) in our sample, and the total scale was 0.69.

**Table 3:** Correlation of mutualism with other motives (Study 1).

	Compassion	Envy	Self-interest	Instrumental Harm	Impartial Beneficence
Mutualism	-0.26***	0.05	0.07	0.13**	0.00
Compassion		-0.21***	0.09	-0.21***	0.29***
Envy			0.06	0.23***	0.11*
Self-interest				-0.03	0.18***
Instrumental Harm					0.23***

*Note*. Effects are standardized regression coefficients [followed by 95% CI].

*** = *p* < .001

** = < .01

* = < .05.

The prediction that mutualism would be negatively associated with utilitarianism was tested with regression analysis. As shown in Table 4, controlling for compassion, envy, and self-interest, we found that no association with mutualism for either Impartial Beneficence (*t(392)* = 0.96, *p* = .336) nor Instrumental Harm (*t(392)* = 1.17, *p* = .243). A further analysis between aspects of mutualism and subscales of utilitarianism also showed the same pattern. Although “Sharing Benefits” (*r* = 0.15, *t(398)* = 2.93, *p* = .003) and “Punishment” (*r* = 0.15, *t(398)* = 2.98, *p* = .002) in Mutualism scale were correlated with Instrumental Harm, the overall results still indicated that most factors of mutualism only have near-zero, non-significant association with utilitarianism. These results confirmed again that neither mutualism nor its component are simply the low end of or opposite conception of Impartial Beneficence, but mutualism did appear to involve a callous element, overlapping weakly with Instrumental Harm.

**Table 4:** Regression models predicting scores of the mutualism scale.

Variables	Model 1	Model 2	Model 3	Model 4
Age	-.01 [-.11 .09]	-.01 [-.11 .09]	-.01 [-.11 .09]	-.01 [-.11 .09]
Sex	-.07 [-.17 .02]	-.08 [-.18 .02]	-.07 [-.16 .03]	-.07 [-.17 .03]
Compassion	-.27 [-.37 -.17] ***	-.28 [-.38 -.17] ***	-.25 [-.35 -.15] ***	-.26 [-.37 -.16] ***
Envy	-.04 [-.14 .07]	-.03 [-.13 .07]	-.03 [-.14 .07]	-.04 [-.14 .07]
Self-interest	.08 [-.02 .18]	.08 [-.02 .18]	.09 [-.01 .19]	.08 [-.01 .18]
Total utilitarianism	.09 [-.01 .19]			
Impartial Beneficence		.07 [-.03 .17]		.05 [-.05 .16]
Instrumental Harm			.08 [-.02 .18]	.06 [-.04 .17]
R^2^	.073	.070	.071	0.071

*Note*. Effects are standardized regression coefficients [followed by 95% CI].

*** = *p* < .001

** = < .01

* = < .05.

## Discussion

In study 1, we developed a scale to measure Mutualism and validate the four component structure of mutualism. We demonstrated that face-valid items chosen on a theory-driven basis form a well-fitting model with a four-factor correlated measurement model, confirming prediction. The scale was reliable, stable, showed construct validity, and demonstrated incremental validity to existing motives, and discriminant validity for existing measures of impartial fairness. The 15-item Mutualism scale consists of items from the four theorised dimensions: Partner Choice, Proportional Sharing of Benefits, Proportional Punishment, and Proportional Helping. These scales predicted subject’s expressed preferences for sharing resources based on contribution in a vignette task. Mutualism scores showed predictive validity for support for redistribution, and incremental validity over and above compassion, malicious envy, and self-interest, had a significant negative relationship with support for redistribution when showing that people with higher mutualism were less willing to share resource compulsively despite personal effort. Mutualism also showed significant negative relationships with support for equal division, believing equal catch division is good and coercing others to share when people were asked to imagine they are living in a small hunting and gathering tribe. This again indicated that people with high mutualism believed those who have more contributions should get more, and they would try to find a new hunting partner if their partner asked a disproportional share.

Discriminant validity for proposed new dimensions is crucial. Despite its being based in concepts of fairness/justice and impartiality we found clear support for independence of mutualism from constructs of Impartial Beneficence (valuing the well-being of everyone equally) and Instrumental Harm (ends-justify-the-means reasoning) underpinning consequentialist ethics– for instance utilitarianism. Our data showed that the Mutualism scale was not strongly related to either dimension of utilitarianism, but suggested an possible link of mutualist sharing and punishment domains to support for instrumental punishment. We wished to test replication of this in study 2. Finally, we successfully replicated previous work on effect of compassion, envy, self-interest, in the presence of mutualism: Jointly, our motivational model accounted for 40% of variance in support for redistribution.

Having demonstrated the reliability and validity of the new Mutualism scale in study 1, in study 2, we set out to test replicability of these findings in a second, independent and pre-registered study, presented next.

## Study 2

In study 2, we set out to replicate each finding from Study 1, verifying the model fit of the four-factor structure to the 15-item Mutualism scale, and replicating each test of criterion, incremental, discriminant, and external validity. All measures were identical to those used in Study 1, and the prediction that our model fit, and validity and reliability findings would replicate directionally and with similar effects sizes were pre-registered on AsPredicted.

## Methods

### Participants

A total of 502 participants were recruited using Prolific Academic (381 females, mean age 36 years (SD = 12.12)), subjects who had joined in Study 1 were not allowed to join in this study. We pre-registered a criterion that subjects who completed the questionnaire less than 30 seconds would be excluded and none of subjects met this criterion. Participants identified as White (n = 468; 93.2%), Mixed (n = 17; 3.4%), Asian (n = 13; 2.6%) and Black (n = 4; 0.8%). Study procedures were approved by the Psychology Research Ethics Committee at the School of Philosophy, Psychology & Language Sciences in the University of Edinburgh (Ref No. 417-1718/6). All participants gave informed consent.

### Measures and procedure

Mutualism was measured with 15-item Mutualism scale. Additional measures for analysing internal and external validity, pragmatic, and incremental predictive validities are as the same as measures we used in Study 1. All items and scales were administered in identical order as previous study. Participants read an Information Sheet and, after giving informed consent joined the study, proceeding to the online items presented via Qualtrics. Scales reliabilities were comparable to study 1 (i.e. Cronbach’s alpha of support for economic redistribution in our sample was 0.90).

## Results

**Fig 4:**
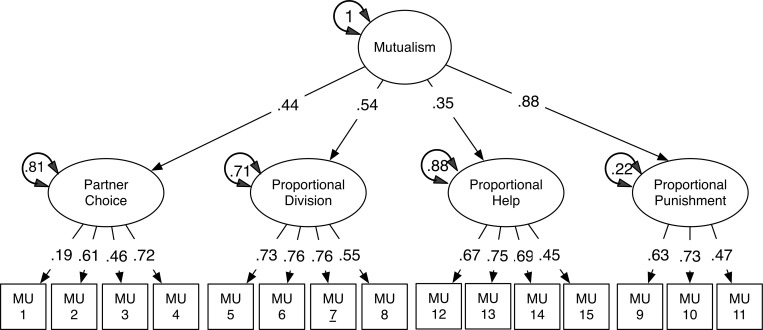
Replication of measurement model of the mutualism scale, Study 2.

To replicate the structural model of Mutualism scale, we conducted a strict confirmatory test, applying the pre-registered 4-factor structural model generated in study 1, with the new data from study 2 to test replication of goodness of fit. The model and standardized parameter estimates are presented in Fig 4.

This direct replication of the 4-factor structural model of the 15-item Mutualism scale found in study 1 showed good fit (χ2 (86) = 184.193, *p* < .000), CFI = .940, RMSEA = .048, and SRMR = 0.065) with fit statistics of the model meeting accepted cut-off criteria [27, 29]. These results thus confirm the findings of Study 1, showing that the exact model of mutualism derived in study 1 has good fit to the observed data of study 2, with comparable item loadings (see Fig 4).

### Reliability and validity

The Mutualism scale showed good internal consistency reliability in study 2 (Cronbach’s alpha of .75 for the total scale and for the subscales: 0.53 (Partner Choice), 0.79 (Proportional Sharing), 0.70 (Proportional Punishment) and 0.66 (Proportional Helping).

**Fig 5:**
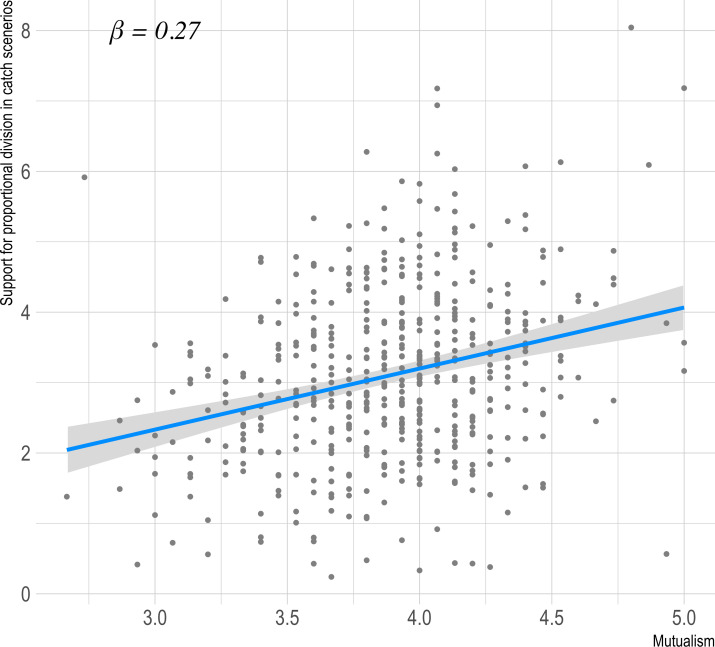
Association of mutualism with proportional catch-sharing scenario sum (Study 2).

We next tested validity using the food sharing scenarios. Results for all three scenarios replicated, with support for equal division showing a significant negative correlation with the Mutualism scale (*r* = -0.23, *t(500)* = -5.21, *p* < .001), as did opposition to the idea that equal catch division is better for the group (*r* = -0.26, *t(500)* = -6.10, *p* < .001) and being unwilling to coerce others to share equally rather than proportionally (*r* = -0.16, *t(500)* = -3.67 *p* < .001). These results have the identical pattern as study 1, indicating again that people with high mutualism tend to avoid sharing what they got equally and have a negative view of equal catch division. The association between Mutualism scale and the sum of three catch-sharing scenarios is shown in Fig 5.

**Fig 6:**
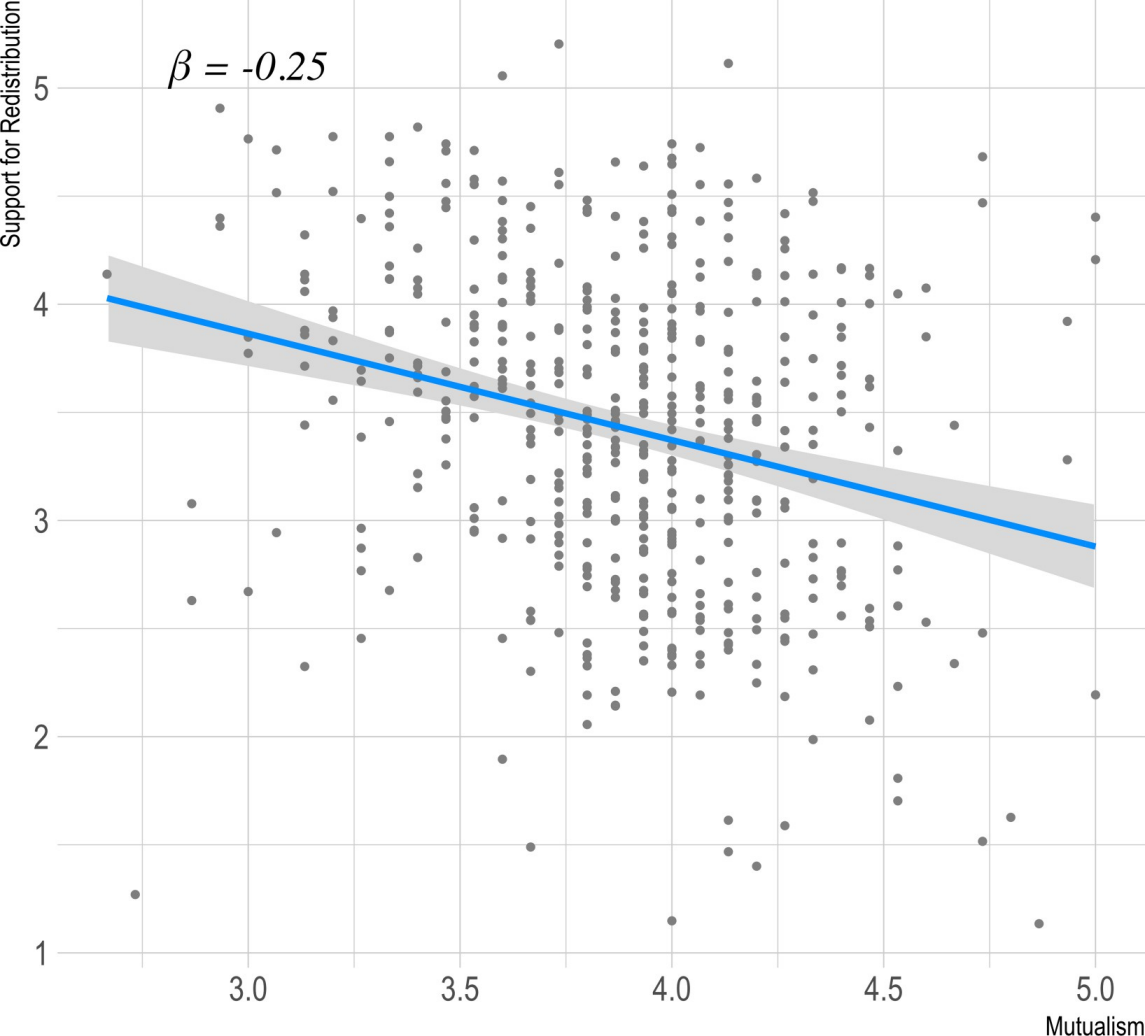
Graph showing effect of mutualism on support for redistribution (Study 2).

External validity against support for redistribution again proved reliable with the negative association of mutualism to support for redistribution replicating with a significant (*t(498)* = -5.93, *p* < .001) effect in the predicted (negative) direction (beta = -0.25, CI95% [-0.34, -0.17]). The relationship between support for redistribution and mutualism is shown in Fig 6.

We next tested replication of the incremental validity of mutualism in predicting support for redistribution over and above compassion, envy, and self-interest by again adding these scales (measured in the same way as in study 1) to the model predicting support for redistribution from mutualism. Mutualism’s independent predictive ability replicated (*t(495)* = -4.75, *p* < .001) in the predicted direction with mutualism scores predicting reduced support for redistribution (beta = -0.16, CI95% [-0.23, -0.09]). As shown in Table 5, effects of compassion, malicious envy, and self-interest also replicated: (*t(495)* = 14.26, *p* < .001; *t(495)* = 8.27, *p* < .001), and *t(495)* = 5.36, *p* < .001, respectively).

**Table 5:** Regression models predicting support for redistribution.

Variables	Model 1	Model 2
Age	-.21 [-.30 -.13] ***	-.02 [-.09 .05]
Sex	-.06 [-.14 .03]	-.13 [-.20 .07] ***
Mutualism	-.25 [-.34 -.17] ***	-.16 [-.23 -.09] ***
Compassion		.51 [.44 .58] ***
Envy		.29 [.22 .36] ***
Self-interest		.18 [.12 .25] ***
R^2^	.106	.441

*Note*. Effects are standardized regression coefficients [followed by 95% CI].

*** = *p* < .001

** = < .01

* = < .05.

Finally, we tested replication of the discriminant validity of mutualism from other scales of fairness in the form of the OUS [23]. As shown in Table 6, controlling compassion, envy and self-interest, mutualism showed no association with Impartial Beneficence (*t(494)* = -1.71, *p* = .088). However, in study 2, the non-significant positive association of mutualism with Instrumental Harm repeated (β = 0.22, CI95% [0.13, 0.31]) and, in study 2, reached significance (*t(494)* = 4.71, *p* < .001). To test the origin of this association, we examined the correlation of each subscale of mutualism with the Instrumental Harm scale, finding that only “Sharing Benefits” (*r* = 0.23, *t(500)* = 5.20, *p* < .001) and “Proportional Punishment” (*r* = 0.18, *t(500)* = 4.10, *p* < .001) had significant correlations with Instrumental Harm. These patterns of association at the sub scale level replicated in the two studies. Therefore, it seems likely that these factors of mutualism either are raised or raise the tendency to view use of force as justifiable to maintain proportional sharing and to punish wrong doers proportionally to their crime. We confirmed also that mutualism had a non-significant association with overall utilitarianism in both study 1 and study 2, indicating the Mutualism scale has good discriminant validity from this construct. In addition, the positive relationship between Instrumental Harm and the two mutualism aspects (“Sharing Benefits” and “Proportional Punishment”) found in study 1 thus replicated, suggesting that mutualism involves an “ends-justify-the-means” element.

**Table 6:** Regression models predicting scores of the mutualism scale.

Variables	Model 1	Model 2	Model 3	Model 4
Age	-.03 [-.12 .06]	-.02 [-.11 .07]	-.02 [-.11 .07]	-.02 [-.11 .07]
Sex	-.10 [-.19 -.01] *	-.10 [-.19 -.02] *	-.09 [-.18 -.01]	-.09 [-.18 -.01] *
Compassion	-.17 [-.26 -.07] ***	-.14 [-.24 -.04] **	-.11 [-.21 -.02] *	-.07 [-.18 .03]
Envy	-.09 [-.19 .00]	-.07 [-.17 .02]	-.10 [-.19 -.01] *	-.09 [-.18 .00]
Self-interest	-.03 [-.11 .06]	-.03 [-.12 .06]	-.03 [-.12 .05]	-.04 [-.12 .05]
Total utilitarianism	.10 [.01 .19] *			
Impartial Beneficence		-.01 [-.10 .09]		-.09 [-.18 .01]
Instrumental Harm			.19 [.11 .28]***	.22 [.13 .31] ***
R^2^	.039	.029	.065	.069

*Note*. Effects are standardized regression coefficients [followed by 95% CI].

*** = *p* < .001

** = < .01

* = < .05.

## Discussion

In this research, we developed and validated scales to measure mutualism. Across two studies, we confirmed that the scale shows the predicted structure of mutualism as four components of Partner Choice, Proportional Sharing, Proportional Punishment, and Proportional Helping. We were able to demonstrate and replicate the test-retest reliability of the scale, replicate the fit of the model, and demonstrate and replicate construct, discriminant, incremental, and external, validity of mutualism scale.

The internal reliability of the scale suggests it will be an informative measure in research, while the stability across a substantial timescale suggests considerable trait stability, and opens the door to developmental studies of mutualism. The finding that scores significantly predicted scenario-based motivation for proportional resource-sharing, for instance attitudes toward equal catch division and coercing others to share validated the prediction that high mutualism motivates ensuring that gains are proportional to contribution. This was further supported by a significant negative association with support for redistribution. The finding that mutualism is an independent construct, overlapping little with compassion, envy and self-interest as well as independence from impartial beneficence help articulate mutualism by showing what it is not, as well validating that effects of mutualism are not reflective of these motives, but rather provide incremental predictive value. The modest association of mutualism with instrumental harm will be valuable to unpack in future research. These findings indicated that the new Mutualism scale can be used to not only predict attitudes in real world, but also distinguish mutualism from existing concepts related to resource distribution.

In building a tool to measure mutualism, we also articulated the relationship among the components of mutualism. Our findings provided clear replication of the predicted four-factor structure, demonstrating strong covariation among the four component traits despite their disparate targets. The research also corroborated the prediction that partner switching itself is a reliable trait, varying between individuals. This provides support expanding the bases of cooperation available beyond tit-for-tat type strategies available to non-partner switching forms of mutualism, and also demonstrates that people are motivated to seek out and defend the freedom to choose partners which underpins these new options for evolving a suite of adaptations for fair cooperation. Understanding variation on this dimension of the mutualist psychological structure will be a fruitful field, with possible application to fields such as politics and economics. Finally, in demonstrating the incremental value of mutualism, the research extended the network of psychological motives underpinning support for redistribution, to include mutualism as an important influence on this central social division, over and above the motivational systems of compassion, envy and self-interest. Future research should examine how mutualism is best incorporated into the three-player model forming the basis for these existing motives [21, 32].

There are multiple potential avenues for research with the scales include attitudes to other predicted policy realms outside redistribution, for instance criminal sentencing, political attitudes, and helping behaviour in public, commercial, and private spheres. Possibilities exist for reframing existing phenomena in terms of partner choice models for instance examining customer loyalty as a manifestation of partner choice. Sociosexual partnerships and mate choice could also be examined, e.g. does mutualism influence short-term versus long-term mating and related characteristic such as divorce? Cross-cultural research and translations of the scale would be valuable. Participants in our research primarily came from the UK, and previous research has shown that perceptions of fairness [34] and levels of consideration to others [35] may be influenced by culture. The scale also makes possible behaviour and molecular genetic research and may help shed light on the evolution of mutualism as well as developmental and cultural effects. To these we would add possible applications for predicting partner switching in response to ill-treatment. Fruitful areas of study present themselves in studies of geographically mobility (for instance movement or willingness to move between jobs, cities, states, and even countries: Mutualists should more readily move in response to ill-treatment. A reviewer suggested that further research on discriminant validity of mutualism from personality traits would be valuable: We agree. For instance, distinguishing mutualism from extraverted sociability or open exploration rather than active partner choice, as well as distinguishing between cooperation based on agreeableness versus mutualist motives. It should be possible also to distinguish mutualist partner choice from a lack of conscientiousness or pathological lack of attachment. Likewise, evolutionary psychological research on whether mutualism influences romantic partner choice would be valuable, as would study of possible interactions of mutualism with valued traits such as skill, intelligence or prestige. Finally, units larger than the individual– corporate bodies such as families, teams, companies, or larger units–might express and differ in mutualism, some endeavouring to limit freedom to switch partners and others actively supporting voluntary collaboration of their constituents and peers.

To conclude, the present studies provide the first psychometric support for mutualism as a coherent complex of helping, punishment, and sharing traits, as well as highlighting the motivation of mutualists to ensure they are free to choose. The studies also provide an open and robust tool to measure mutualism in future research and applications. The data providing strong evidence for the reliability and validity of this scale. Mutualism is a potentially central social trait with applications in areas as disparate as moral theory, political policy and affiliation, and economics, but it has lacked measures making it amenable to study. This research fills a gap between theory and practice and provides a valid but brief tool for researchers to apply.

## Supporting information

S1 Appendix Details of research material, data, analysis and pre-registrations. (DOCX)
