# Supplementary material for: Free to choose: Mutualist motives for partner choice, proportional division, punishment, and help
Source: PLoS One. 2022 May 5;17(5):e0266735. doi: 10.1371/journal.pone.0266735 (PMC9071132; doi:10.1371/journal.pone.0266735)
Supplement: S1 Appendix — (DOCX) [file pone.0266735.s001.docx]

**Pre-registrations**

We report how we determined our sample size, all data exclusions (if any), all manipulations, and all measures in the study.

All studies reported in this article were formally pre-registered at AsPredicted

Study 1: <https://aspredicted.org/blind.php?x=ai28mw>

Study 2: <https://aspredicted.org/blind.php?x=dy2ci3>

All questionnaires and all data are available at <https://osf.io/8zay2/?view_only=76adabe7f3624c8da10560e9843f3b27>
